# Valence State Manipulation of Cerium Oxide Nanoparticles on a Titanium Surface for Modulating Cell Fate and Bone Formation

**DOI:** 10.1002/advs.201700678

**Published:** 2017-12-18

**Authors:** Jinhua Li, Jin Wen, Bin Li, Wan Li, Wei Qiao, Jie Shen, Weihong Jin, Xinquan Jiang, Kelvin W. K. Yeung, Paul K. Chu

**Affiliations:** ^1^ Department of Orthopaedics and Traumatology Li Ka Shing Faculty of Medicine The University of Hong Kong Pokfulam Hong Kong 999077 China; ^2^ Department of Physics Department of Materials Science and Engineering City University of Hong Kong Tat Chee Avenue Kowloon Hong Kong 999077 China; ^3^ Shenzhen Key Laboratory for Innovative Technology in Orthopaedic Trauma Department of Orthopaedics and Traumatology The University of Hong Kong‐Shenzhen Hospital Shenzhen 518053 China; ^4^ Department of Prosthodontics Ninth People's Hospital affiliated to Shanghai Jiao Tong University School of Medicine Shanghai 200011 China; ^5^ Department of Orthopedics The First Affiliated Hospital of Zhengzhou University Zhengzhou 450052 China; ^6^ Dental Materials Science Applied Oral Sciences Faculty of Dentistry The University of Hong Kong Hong Kong 999077 China

**Keywords:** cerium oxide nanoparticles, immune response, macrophages, osteogenic differentiation, stem cells

## Abstract

Understanding cell–biomaterial interactions is critical for the control of cell fate for tissue engineering and regenerative medicine. Here, cerium oxide nanoparticles (CeONPs) are applied at different Ce^4+^/Ce^3+^ ratios (i.e., 0.46, 1.23, and 3.23) to titanium substrate surfaces by magnetron sputtering and vacuum annealing. Evaluation of the cytotoxicity of the modified surface to cultured rat bone marrow mesenchymal stem cells (BMSCs) reveals that the cytocompatibility and cell proliferation are proportional to the increases in Ce^4+^/Ce^3+^ ratio on titanium surface. The bone formation capability induced by these surface modified titanium alloys is evaluated by implanting various CeONP samples into the intramedullary cavity of rat femur for 8 weeks. New bone formation adjacent to the implant shows a close relationship to the surface Ce^4+^/Ce^3+^ ratio; higher Ce^4+^/Ce^3+^ ratio achieves better osseointegration. The mechanism of this in vivo outcome is explored by culturing rat BMSCs and RAW264.7 murine macrophages on CeONP samples for different durations. The improvement in osteogenic differentiation capability of BMSCs is directly proportional to the increased Ce^4+^/Ce^3+^ ratio on the titanium surface. Increases in the Ce^4+^/Ce^3+^ ratio also elevate the polarization of the M2 phenotype of RAW264.7 murine macrophages, particularly with respect to the healing‐associated M2 percentage and anti‐inflammatory cytokine secretion. The manipulation of valence states of CeONPs appears to provide an effective modulation of the osteogenic capability of stem cells and the M2 polarization of macrophages, resulting in favorable outcomes of new bone formation and osseointegration.

## 1 Introduction

Understanding the interactions between stem cells and biomaterials surface is essential for controlling stem cell fates, including adhesion, proliferation, and osteogenic differentiation, in bone tissue engineering and regenerative medicine.^1^ Tremendous advancements have been made in tailoring bone biomaterials to modulate stem cell fates, based on two general strategies: (1) modulation of the physical and chemical properties of biomaterial surfaces,^2^ and (2) incorporation of bioactive cues onto biomaterial surfaces.^3^ For instance, electroactive biomaterials have been designed to stimulate the osteogenesis of stem cell, thereby inducing subsequent bone regeneration. As inspired by the piezoelectric property of bones, Yu et al. designed the microscale piezoelectric zones (MPZs) with the use of K_0.5_Na_0.5_NbO_3_ ceramics to guide the stem cell osteogenic differentiation in vitro and in vivo.^4^ Indeed, when an inverse piezoelectric effect was introduced to bone, the strain induced in bone matrix could stimulate the cellular activity of bone cells and then improved bone regeneration.^5^ When low‐intensity pulsed ultrasound (LIPUS) was applied to porous titanium alloy scaffold, the micromechanical strain induced by LIPUS would therefore initiate osteoblastic differentiation, bone formation and maturation as well as bony ingrowth in the porous scaffold.^6^ A recent study by Ning et al. proposed the use of periodic microscale electric field (MEF) to induce bone‐implant integration. Two parallel semiconducted anatase and rutile TiO_2_ zones were established on titanium implant and this electrical cue was able to direct stem cell osteogenic differentiation and bone regeneration on implant surface.^7^ However, identification of the influences of specific surface features of biomaterials on stem cell functions remains difficult due to the substrate complexity, and the mechanism underlying their interaction is still not well understood. Nonetheless, macrophages appear to play a critical role in host reactions in the early stage of bone biomaterial insertion, and their initial response to a biomaterial can determine the success of biomaterial‐associated osteogenesis.[[qv: 1b,8]]

In response to various tissue microenvironmental cues, highly plastic macrophages can alter their phenotypes and functions to display the classically activated proinflammatory M1 and alternatively activated antiinflammatory M2 forms.^9^ The prohealing M2‐polarized macrophage phenotype can elicit positive outcomes on osteogenesis, angiogenesis, and osseointegration,[[qv: 8a,10]] and macrophage polarization can be modulated by the surface physics and chemistry cues of bone biomaterials. Therefore, modulation of the macrophage polarization response to biomaterials may be a promising strategy for eliciting favorable outcomes for bone tissue engineering and regenerative medicine.^11^

In fact, as a foreign body, a biomaterial implant is recognized by the host immune system and triggers an immune reaction that may eventually determine the in vivo fate of the bone biomaterials.[[qv: 11f,12]] Introduction of an implant into the body initiates an inflammatory cascade due to cell and tissue damage, and this cascade persists for roughly 4 d.^13^ This early inflammatory response is beneficial to the host, but termination of subsequent inflammation is critical for blocking tissue damage and promoting tissue regeneration.^14^ Therefore, an in‐depth understanding of the mechanism underlying the immune response mediated by a bone biomaterial would aid in the development of novel bone biomaterials that can create a favorable local immune environment for optimal osteogenesis and osseointegration.

Recently, cerium oxide nanoparticles (CeONPs) have attracted increasing interest for biological applications because of their excellent catalytic activities, which arise from quick and expedient switches in oxidation state between Ce^4+^ (CeO_2_) and Ce^3+^ (Ce_2_O_3_) during redox reactions.^15^ In particular, their capability to quench free radicals (i.e., antioxidant activity) give CeONPs great potential for use in therapy of diseases caused by reactive oxygen species (ROS), such as retinal degeneration,^16^ cardiovascular pathology,^17^ neurodegenerative disorders,^18^ and spinal cord injury.^19^ Most of the CeONP characteristics are endowed by the co‐existence of both Ce^4+^ and Ce^3+^ oxidation states. In addition, nano‐CeO_2_ regions promote cell proliferation on a functionalized polymer scaffold surface, whereas nano‐Ce_2_O_3_ regions have an inhibitory effect on cell proliferation.^20^ CeO_2_ nanoparticles can also induce aligned growth of stem cells and improve the bioactivity of polymer scaffold surfaces.^21^ They also show favorable biocompatibility and exert a protective effect on normal cells even at levels that exhibit antitumor effects on cancer cells.^22^

This mixed valence state of CeONPs has therapeutic potential due to the possibility of scavenging ROS in cells.^16^, ^23^ Previous in vitro studies have shown that CeONPs can react catalytically with superoxide and hydrogen peroxide to mimic the biological actions of superoxide dismutase (SOD)[[qv: 23b,24]] and catalase.^25^ At physiological pH, in particular, Ce^3+^ and Ce^4+^ sites on CeONP surfaces show this SOD and catalase mimetic activity, respectively.^15^ For these reasons, CeONPs supplied to murine J774A.1 macrophages can scavenge ROS and inhibit inflammatory mediator production.^26^ These significant advantages of CeONPs support the speculation that CeONPs could be useful in modifying bone biomaterials to impart immunomodulatory characteristics that will allow regulation of macrophage behavior and promotion of stem cell osteogenic differentiation and bone tissue regeneration.

Nevertheless, few designs that have immobilized CeONPs onto bone biomaterials have also demonstrated immunomodulatory and osteogenic‐inducing activity. Titanium‐based biomaterials are widely applied in orthopedic and dental implants due to their desirable biocompatibility and mechanical properties.^27^ However, their bioactivity is insufficient in terms of bone‐implant osseointegration and fibrotic scarring mediated by macrophages is usually found during foreign body response.^28^ Therefore, the aim of this study was to design a functional titanium surface that is able to modulate the macrophage immune response and the osteogenic capability of stem cells, followed by stimulation of bone tissue regeneration. A promising strategy to achieve this goal would appear to be modulation of the Ce valence states of extracellular CeONPs.

The biofunction of Ce^4+^ and Ce^3+^ particles is size dependent, and the fraction of Ce^4+^ in particles generally increases with increases in the particle size.^29^ Hence, a magnetron sputtering technique was adopted to control the particle size and the Ce^4+^/Ce^3+^ ratio of CeONPs was optimized by deposition time. We hypothesized that this functional surface with its higher Ce^4+^/Ce^3+^ ratio can effectively modulate macrophage behavior, thereby promoting new bone formation. Following characterization of the physical and chemical properties of the CeONP‐modified titanium surface, the cytocompatibility of this surface was tested by culturing rat bone marrow mesenchymal stem cells (BMSCs), with subsequent in vivo animal implantation for osteogenic capability assessment. The detailed biological responses and underlying mechanism of new bone formation were investigated by culturing, rat BMSCs and RAW264.7 macrophages, respectively, onto the CeONP‐modified surface.

## 2 Results

### 2.1 Materials Characterization

**Figure 1:**
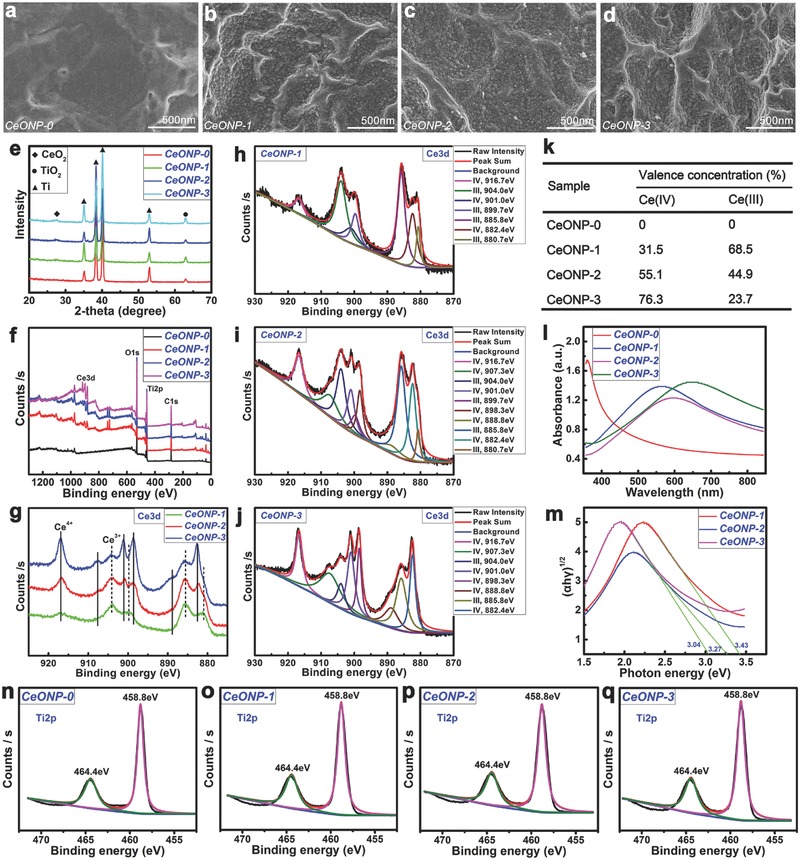
Surface morphologies of samples CeONP‐0 a), CeONP‐1 b), CeONP‐2 c), and CeONP‐3 d). XRD patterns e) and XPS full spectra f) of the samples. Development of Ce 3d XPS spectra f) and the fitted Ce 3d spectra of CeONP‐1 h), CeONP‐2 i), and CeONP‐3 j), with the corresponding analysis results k) of valence concentration of Ce(IV) and Ce(III). UV–vis absorbance spectra l) of the samples and the corresponding conversion curves using the Kubelka–Munk function m). Fitted high resolution XPS spectra of Ti2p core levels for the samples CeONP‐0 n), CeONP‐1 o), CeONP‐2 p), and CeONP‐3 q).

**Figure** **1**a–d shows the surface morphologies of various samples. Acid cleaning left a relatively flat topography on the CeONP‐0 surface (Figure 1a). Magnetron sputtering (2 min) and vacuum annealing then revealed tiny homogeneously distributed nanoparticles on the CeONP‐1 surface (Figure 1b). Increasing the deposition time to 3 min (Figure 1c) and 5 min (Figure 1d) caused these nanoparticles to develop gradually into relatively larger ones. The X‐ray diffractometry (XRD) patterns (Figure 1e) and the diffraction peaks of the titanium substrate revealed a diffraction peak for the CeO_2_ (111) facet.^21^ In addition, a peak of TiO_2_ emerged due to the crystallization of a natural oxide layer on the titanium surface.^30^ Therefore, these nanoparticles had the characteristics of CeO_2_ nanoparticles.

Figure 1f shows the surface X‐ray photoelectron spectroscopy (XPS) survey spectra of various samples. The corresponding surface elemental compositions are listed in **Table** **1**. Increases in the deposition time from 2 to 5 min increased the content of the Ce element on the surface from an initial 0 at% for CeONP‐0, 3.57 at% for CeONP‐1, and 5.82 at% for CeONP‐2 to 7.58 at% for CeONP‐3. Accordingly, the content of the Ti element on the surface decreased from 25.53 at% for CeONP‐0, 25.50 at% for CeONP‐1, and 21.31 at% for CeONP‐2 to 17.13 at% for CeONP‐3. Interestingly, the content of the surface O element dropped from 74.47 at% for CeONP‐0 to 70.93 at% for CeONP‐1, and then increased to 72.87 at% for CeONP‐2 and 75.29 at% for CeONP‐3.

**Table 1:** Elemental components of CeONP‐0, CeONP‐1, CeONP‐2, and CeONP‐3 analyzed by XPS. Analyzing area: 2.0 mm × 2.0 mm

Sample	Elemental concentration [at%]		
	Ce	Ti	O
CeONP‐0	0	25.53	74.47
CeONP‐1	3.57	25.50	70.93
CeONP‐2	5.82	21.31	72.87
CeONP‐3	7.58	17.13	75.29

The high resolution XPS spectra of Ce 3d were acquired from the surfaces of various CeONP samples, as shown in Figure 1g; this consisted of two parts: (i) 916.7, 907.3, and 901.0 eV for Ce 3d_3/2_ and 898.3, 888.8, and 882.4 eV for Ce 3d_5/2_ of CeO_2_ (solid line); (ii) 904.0 and 899.7 eV for Ce 3d_3/2_ and 885.8 and 880.7 eV for Ce 3d_5/2_ of Ce_2_O_3_ (dashed line).^20^ The evolution of the Ce 3d XPS spectra was analyzed by further fitting of these high resolution spectra, as shown in Figure 1h–j. A detailed description of the Ce 3d spectra fitting procedure was given previously.^31^ Figure 1h–j shows qualitatively the relative changes in the Ce 3d XPS spectra for various CeONP samples. The corresponding quantitative results of the valence concentrations shown in Figure 1k confirm that, as deposition time increased, the concentrations of surface Ce^4+^ (CeO_2_) increased from 0% for CeONP‐0, 31.5% for CeONP‐1 and 55.1% for CeONP‐2 to 76.3% for CeONP‐3. Accordingly, the concentrations of surface Ce^3+^ (Ce_2_O_3_) decreased from 68.5% for CeONP‐1 to 23.7% for CeONP‐3.

Figure 1i depicts the UV–vis absorption spectra of the various samples. A strong absorption at ≈400 nm for CeONP‐0 was ascribed to the absorption edge of TiO_2_. Interestingly, the increase in deposition time from 2 to 5 min shifted the absorption edge to a longer wavelength range. The diffuse reflectance spectra were converted into equivalent absorption coefficients using the Kubelka–Munk function:^32^ α = (1 − *R*)^2^/2*R*, *αhν* = *C*_1_(*hν* – *E*_g_)^2^, *hν* = 1240/λ, where α is the optical absorption coefficient near the absorption edge for the indirect interband transition, *R* is the reflectance of the semiconductor, *C*_1_ is a constant for the indirect transition, *hν* is the photon energy, *E*_g_ is the indirect bandgap energy (eV), and λ is the wavelength (nm). Figure 1m depicts the (*αhν*)^1/2^ plot versus *hν*; here, the vertical segments of the spectra were extended to intersect with the hν axis to obtain the *E*_g_ for the CeONP samples. The narrowed bandgap agreed with the red shift of the absorption edge, which corresponded to the increase in nanoparticle size and surface Ce^4+^ content for the CeONP samples from 2 to 5 min.^33^ In addition, the high resolution XPS spectra of Ti2p core levels from the various samples were fitted in Figure 1n–q. The doublet peak at 458.8 eV and 464.4 eV belongs to the Ti2p_3/2_ and Ti2p_1/2_ in TiO_2_, respectively.^30^ This indicates that the TiO_2_ remained on titanium substrate after depositing cerium oxides.

### 2.2 Cell Viability

**Figure 2:**
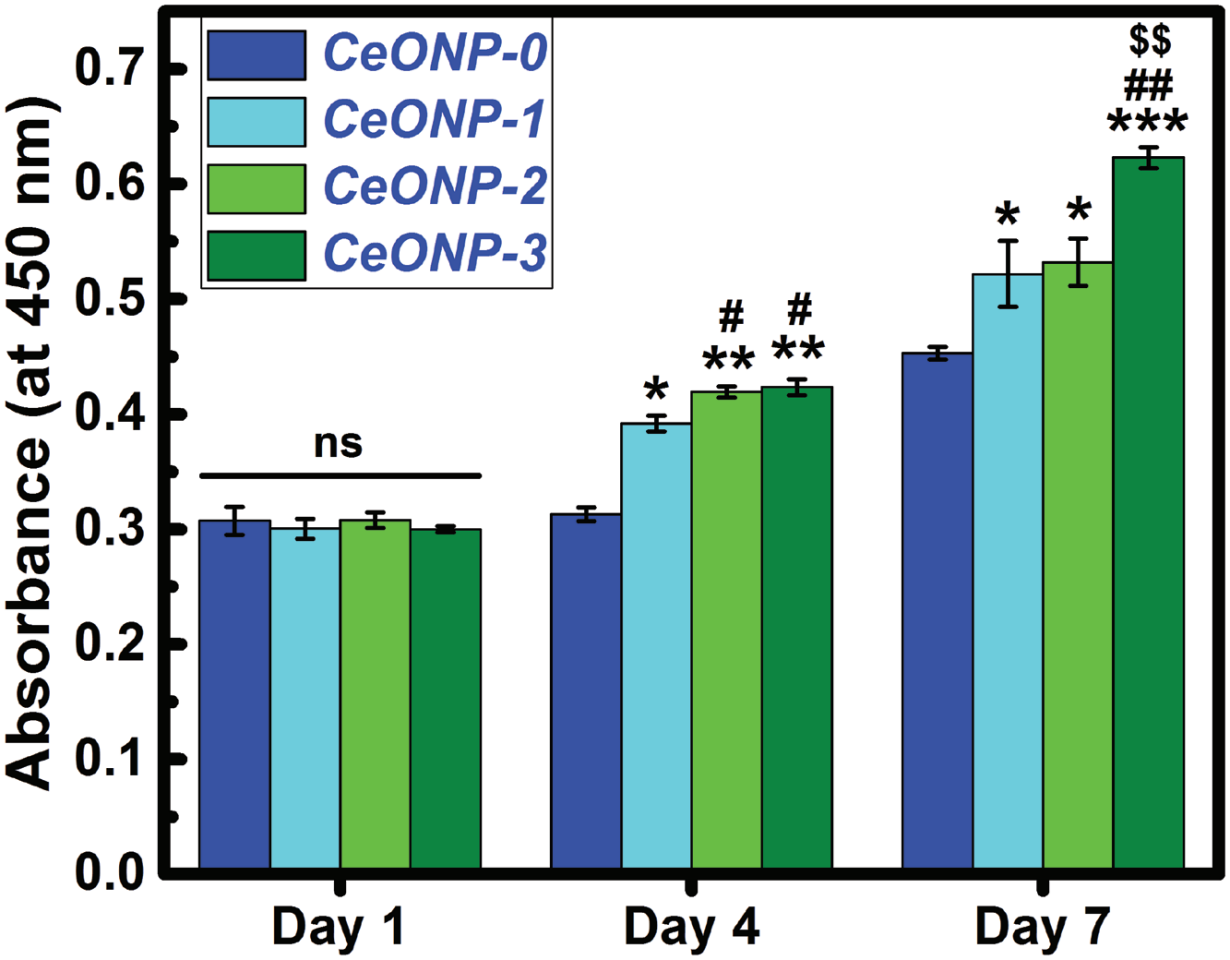
CCK‐8 assay results of proliferation and viability of rat BMSCs after 1, 4, and 7 d of culture on samples CeONP‐0, CeONP‐1, CeONP‐2, and CeONP‐3. Note: *P < 0.05, **P < 0.01, ***P < 0.001 versus CeONP‐0; ^#^ *P* < 0.05, ^##^ *P* < 0.01 versus CeONP‐1; ^$$^ *P* < 0.01 versus CeONP‐2; ns, not significant.

**Figure** **2** shows the proliferation and viability of rat BMSCs after culturing on various samples for 1, 4, and 7 d. At day 1, no significant difference was noted among the groups. However, after 4 d of culture, obvious differences emerged between the CeONP samples and the control group. The CeONP samples significantly promoted cell proliferation on the surface in a manner dependent on the Ce^4+^/Ce^3+^ ratio (0.46 for CeONP‐1, 1.23 for CeONP‐2, and 3.23 for CeONP‐3). Furthermore, the increase in the surface Ce^4+^ content resulted in a better promotion effect on cell proliferation for the CeONP‐2 and CeONP‐3 samples than for the CeONP‐1 samples (*P* < 0.05). At day 7, the cell growth on all samples maintained an upward trend, with an apparent difference between the testing groups and the control group. CeONP‐3 induced the highest cell proliferation and viability. Thus, an increase in the surface Ce^4+^ content can promote the proliferation of rat BMSCs on CeONP samples.

### 2.3 In Vivo Osseointegration

#### 2.3.1 Micro‐CT Evaluation of Bone Formation

**Figure 3:**
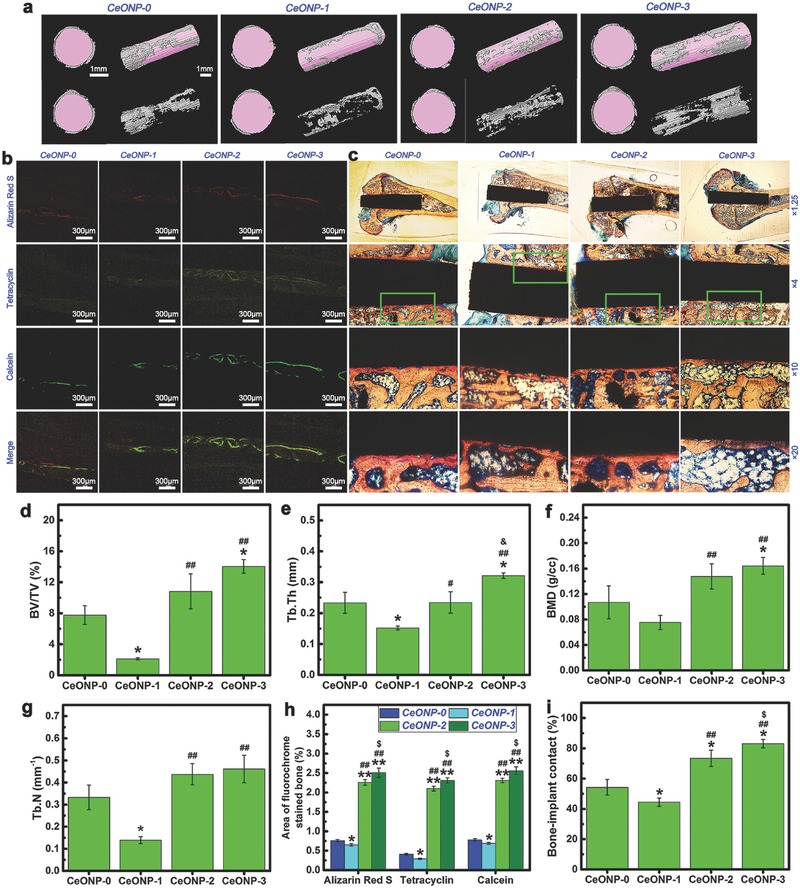
Micro‐CT images of reconstructed 3D models of surrounding bones in transverse and longitudinal views, with or without implants a), accompanied by the corresponding quantitative analysis results of BV/TV d), Tb.Th e), BMD f), and Tb.N g). Sequential fluorescent labeling observation b), accompanied by the corresponding analysis results h) of the area of bone stained with the three fluorochromes. (Note: Red labeling, Alizarin Red S, week 2; yellow labeling, tetracycline, week 4; green labeling, calcein, week 6; *P < 0.05, **P < 0.01 versus CeONP‐0; ^##^ *P* < 0.01 versus CeONP‐1; ^$^ *P* < 0.05 versus CeONP‐2.) Histological observation c) of the CeONP‐0, CeONP‐1, CeONP‐2, and CeONP‐3 sections stained with Van Gieson's picrofuchsin, accompanied by the corresponding analysis results i) of bone‐implant contact from the histomorphometric measurement at ×10 magnification. (Note: *P < 0.05 versus CeONP‐0; ^##^ *P* < 0.01 versus CeONP‐1; ^$^ *P* < 0.05 versus CeONP‐2.)

All the samples were implanted in rat femur bones for 8 weeks to enable the in vivo evaluation of osseointegration. **Figure** **3**a shows the reconstructed micro‐CT images along the central axis of the implants, with the implants marked in pink and the newly formed bones marked in grey. This figure shows that the bone volume is visually larger around the surface of the CeONP‐3 implant than around the other implants. As shown in Figure 3d, the BV/TV of CeONP‐3 was 14.1%, which was significantly higher than the 7.8% obtained with CeONP‐0 (*P* < 0.05) and 2.1% with CeONP‐1 (*P* < 0.01). CeONP‐2 also had a better outcome of 10.8% compared with CeONP‐1 (*P* < 0.01). A similar trend was observed for Tb.Th (Figure 3e) and bone mineral density (BMD) (Figure 3f). Both CeONP‐3 (0.46 mm^−1^) and CeONP‐2 (0.44 mm^−1^) produced higher outcomes than CeONP‐1 (0.14 mm^−1^, *P* < 0.01) for Tb.N (Figure 3g). For these parameters, worse outcomes were obtained with CeONP‐1 than with CeONP‐0 (*P* < 0.05). Consequently, the new bone formation around the implants displayed an obvious correlation with the surface Ce^4+^ content in the CeONPs.

#### 2.3.2 Sequential Fluorescent Labeling

The process of new bone formation and mineralization around the implants was recorded using three types of fluorochrome at different time points and the results are shown in Figure 3b. This figure shows that CeONP‐3 and CeONP‐2 had better outcomes in terms of promoting new bone formation when compared with CeONP‐0 and CeONP‐1. The percentages of fluorochrome labeled area are shown in Figure 3h. At week 2, the percentage of Alizarin Red S labeled area (red) was significantly higher for CeONP‐2 and CeONP‐3 than for CeONP‐0 and CeONP‐1 (*P* < 0.01). CeONP‐1 had a lower value than CeONP‐0 (*P* < 0.05) and CeONP‐3 had a better outcome than CeONP‐2 (*P* < 0.05). At week 4 and week 6, the percentages of tetracycline hydrochloride labeled area (yellow) and calcein labeled area (green) exhibited a similar tendency. Therefore, these results confirmed that the increase in surface Ce^4+^ content in the CeONPs could promote new bone formation and mineralization at the bone‐implant interface.

#### 2.3.3 Histological Observation

The van Gieson's picrofuchsin staining is a central histological test for establishing whether implants are in direct contact with the surrounding bones. The histological staining results for the sections in the present study are shown in Figure 3c. The corticocancellous site shows a close apposition of bone to implant for CeONP‐3. The implant surface bonds tightly and directly with the newly formed bone, virtually without fibrous or connective tissue that would prevent its direct contact with new bone. Similarly, CeONP‐2 also produces a good outcome regarding new bone apposition. By contrast, only a small amount of new bone appeared around the CeONP‐1 implant within the corticocancellous bone, and an interspace was apparent between the newly formed bone and the implant surface. The percentages of bone‐implant contact (BIC) in the osseointegration region, observed at ×10 magnification, are given in Figure 3i. In agreement with the micro‐CT results, significantly higher BIC percentages were obtained with CeONP‐3 and CeONP‐2 than with CeONP‐0 (*P* < 0.05) and CeONP‐1 (*P* < 0.01). The BIC percentage was also lower for CeONP‐1 than for CeONP‐0 (*P* < 0.05) and the BIC percentage was higher for CeONP‐3 than for CeONP‐2 (*P* < 0.05). Therefore, an increase in the surface Ce^4+^ content in the CeONPs can promote new bone formation around the implant and enhance osseointegration.

#### 2.3.4 Biomechanical Push‐Out Test

**Figure 4:**
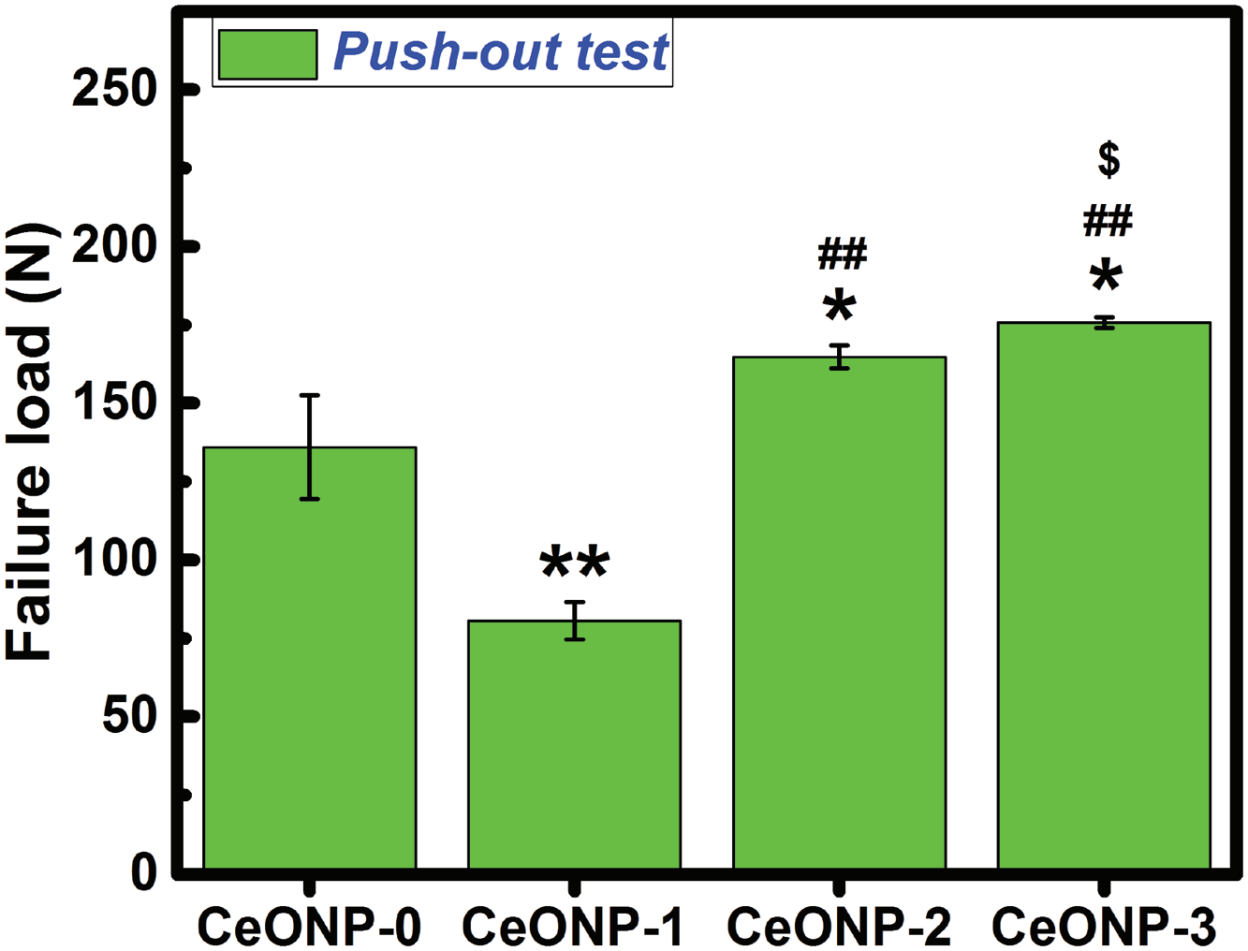
Results of biomechanical testing. Note: *P < 0.05, **P < 0.01 versus CeONP‐0; ^##^ *P* < 0.01 versus CeONP‐1; ^$^ *P* < 0.05 versus CeONP‐2.

The biomechanical push‐out test was utilized to evaluate the quality of osseointegration of the implants. **Figure** **4** shows that, among the various groups, CeONP‐3 had the largest failure load of 175.7 N whereas CeONP‐1 had the lowest failure load of 80.6 N. Both CeONP‐3 and CeONP‐2 (164.7 N) had larger failure loads than CeONP‐0 (135.9 N, *P* < 0.05) and CeONP‐1 (*P* < 0.01). The failure load was lower for CeONP‐1 than for CeONP‐0 (*P* < 0.01). Therefore, an increase in the surface Ce^4+^ content on CeONPs could enhance the osseointegration quality of the implant.

#### 2.3.5 Scanning Electron Microscopy (SEM) Observation

**Figure 5:**
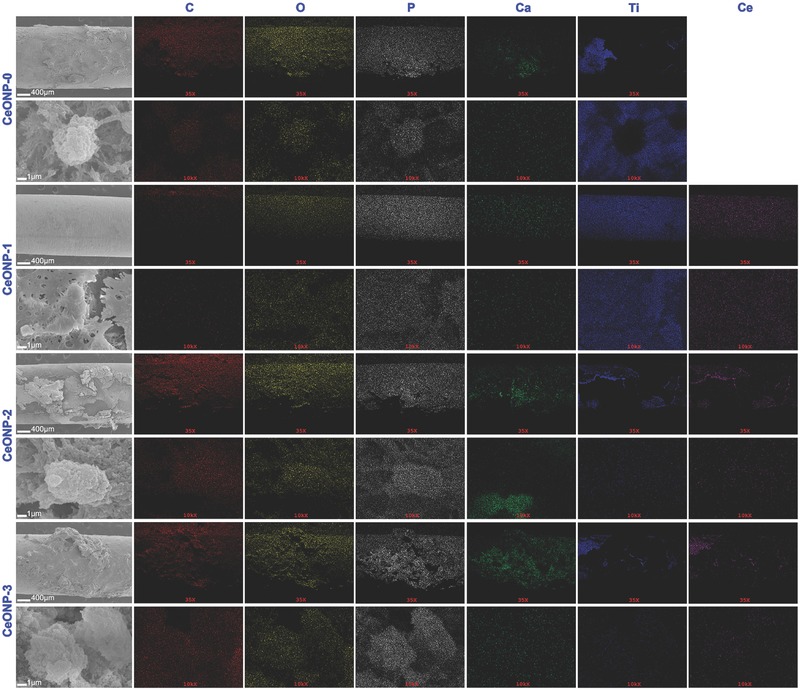
Surface morphologies of the implants of various samples, accompanied by the corresponding EDS elemental mapping and distribution of C, O, P, Ca, Ti, and Ce.

The surface morphologies of the pushed‐out implants were observed by SEM and the element distributions in the implants were mapped by energy dispersive X‐ray spectroscopy (EDS) for further examination of the new bone formation on the surface of the implants in the bone marrow cavity. The SEM images in **Figure** **5** show the typical interface bonding status between the implants and new bone tissues. The CeONP‐0, at low magnification, shows some large blocks of new bone tissues remaining on the implant surface, indicating a strong interface bonding strength between the implant and new bone tissues. At high magnification, the implant showed a rough surface due to the adhesion of new bone tissues (Figure S1, Supporting Information). This rough structure was more apparent at the higher magnification in Figure 5. By contrast, CeONP‐1, shows a relatively bare implant surface, indicating poor interface bonding strength between the implant and new bone tissues. At high magnification, the implant displayed a relatively smooth surface morphology (Figure S1, Supporting Information) and this became more obvious at higher magnification, as shown in Figure 5. CeONP‐2 and CeONP‐3 show larger blocks of new bone tissues remaining on the implant surface, indicating a very strong interface bonding strength between the implant and new bone tissues. This was further supported by the rough morphology seen under high magnification. Therefore, the SEM results were agreed well with the push‐out results in Figure 4.

EDS mapping showed that the newly formed bone tissues were characterized by intense distribution maps of calcium (green) and phosphorus (gray). Figure 5 shows the mapping results that were very consistent with the SEM results. The weak distribution maps for titanium (blue), as a counterpart, can be used to estimate the coverage and thickness of new bone tissues on the implants. Therefore, the SEM and EDS results agreed well with the results for new bone formation obtained with the other methods.

### 2.4 Osteogenic Mechanism

#### 2.4.1 In Vitro Osteogenic Regulation

**Figure 6:**
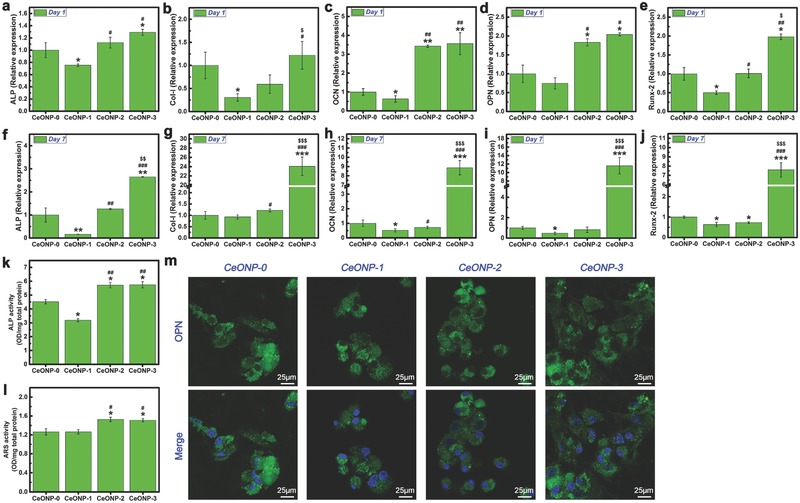
Expression levels of osteogenic‐related genes ALP a,f), Col‐I b,g), OCN c,h), OPN d,i), and Runx‐2 e,j) by real time PCR after culturing rat BMSCs on various samples for 1 and 7 d. ALP activity of rat BMSCs after 7 d of culture on various samples k). ARS activity of rat BMSCs after 14 d of culture on various samples l). Detection of OPN expression after culturing rat BMSCs on the samples for 7 d by immunofluorescence assay m). Note: *P < 0.05, **P < 0.01, ***P < 0.001 versus CeONP‐0; ^#^ *P* < 0.05, ^##^ *P* < 0.01, ^###^ *P* < 0.001 versus CeONP‐1; ^$^ *P* < 0.05, ^$$^ *P* < 0.01, ^$$$^ *P* < 0.001 versus CeONP‐2.

Further understanding of the outcome of new bone formation and mineralization and their regulation by CeONPs was obtained using quantitative real‐time polymerase chain reaction (PCR) assays. The key osteogenic‐related markers, including alkaline phosphate (ALP) (**Figure** **6**a,f), collagen type I (Col‐I) (Figure 6b,g), osteocalcin (OCN) (Figure 6c,h), osteopontin (OPN) (Figure 6d,i), and runt‐related transcription factor 2 (Runx‐2) (Figure 6e,j), were investigated to determine the influence of the surface Ce^4+^ content of the CeONPs on the osteogenic differentiation of rat BMSCs at the molecular level. The cells were cultured on CeONPs for 1 and 7 d. At day 1 (Figure 6a–e), a significant promoting effect was observed for the expression of the ALP, Col‐I, OCN, OPN, and Runx‐2 genes in response to CeONP‐3 and CeONP‐2 but not in response to CeONP‐1 and CeONO‐0 (*P* < 0.05). CeONP‐1 induced an obvious down‐regulation of these osteogenesis genes when compared with CeONP‐0 (*P* < 0.05). At day 7 (Figure 6f–j), the difference in the expression of these genes between CeONP‐3 and other groups became more significant (*P* < 0.01).

ALP is an early marker of BMMSC differentiation, so its measurement at day 7 was used to evaluate the osteogenic differentiation potential of the CeONP samples. As shown in Figure 6k, significantly higher ALP activity was observed for both CeONP‐3 and CeONP‐2 than for CeONP‐1 (*P* < 0.01) and CeONP‐0 (*P* < 0.05). ALP activity was also lower for CeONP‐1 than for CeONP‐0 (*P* < 0.05).

Similarly, Alizarin Red S (ARS) was determined to evaluate calcium nodule formation in the samples. As shown in Figure 6l, ARS activity was apparently higher for CeONP‐3 and CeONP‐2 than for CeONP‐0 and CeONP‐1 (*P* < 0.05).

OPN expression in the rat BMSCs was detected by an immunofluorescence assay with DyLight 488. As shown in Figure 6m, CeONP‐3 induced the strongest immunofluorescence labeling of OPN expression, whereas CeONP‐1 produced the weakest green fluorescence. More BMSCs that expressed the relevant specific protein were detected for CeONP‐3 than for the other groups. Therefore, an increase in the surface Ce^4+^ content in the CeONPs can promote in vitro osteogenic differentiation of rat BMSCs, thereby accounting, at least in part, for the enhanced new bone formation around the implant and for osseointegration.

#### 2.4.2 Macrophage Response

**Figure 7:**
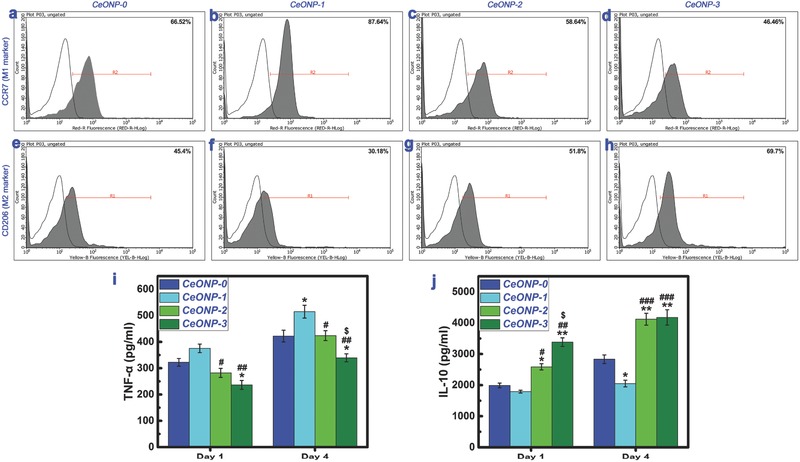
Expression of cell surface markers on RAW264.7 macrophages determined by flow cytometry, showing the percentages of M1 phenotype (CCR7, a–d) and M2 phenotype (CD206, e–h). Production of cytokines TNF‐α i) and IL‐10 j) secreted by RAW264.7 macrophages cultured on various samples determined by ELISA. Note: *P < 0.05, **P < 0.01 versus CeONP‐0; ^#^ *P* < 0.05, ^##^ *P* < 0.01, ^###^ *P* < 0.001 versus CeONP‐1; ^$^ *P* < 0.05 versus CeONP‐2.

**Figure** **7** shows the analysis results for flow cytometry assay and ELISA of RAW264.7 macrophages. The percentage of M1 cells that expressed the surface marker CCR7 showed the following trend (Figure 7a–d): CeONP‐1 (87.64%) > CeONP‐0 (66.52%) > CeONP‐2 (58.64%) > CeONP‐3 (46.46%). By contrast, the percentage of M2 cells expressing the surface marker CD206 showed the following trend (Figure 7e–h): CeONP‐1 (30.18%) < CeONP‐0 (45.40%) < CeONP‐2 (51.80%) < CeONP‐3 (69.70%). The production of the pro‐inflammatory cytokine tumor necrosis factor‐α (TNF‐α) in the supernatants recovered from various samples (Figure 7i) indicated a significantly higher TNF‐α concentration for CeONP‐1 than for CeONP‐3 (*P* < 0.01) and CeONP‐2 (*P* < 0.05) at day 1.The TNF‐α concentration was also lower in response to CeONP‐3 than in response to CeONP‐0 (*P* < 0.05). The same trend was observed after 4 d of culture. A statistically significant difference was observed between the responses to CeONP‐3 and CeONP‐2 (*P* < 0.05), and the concentration was higher for CeONP‐1 than for CeONP‐0 (*P* < 0.05).

Production of the anti‐inflammatory cytokine interleukin‐10 (IL‐10) in the medium retrieved from various samples (Figure 7j) indicated higher expression of IL‐10 in response to CeONP‐3 and CeONP‐2 when compared with CeONP‐0 and CeONP‐1, and this difference was statistically significant at both day 1 (*P* < 0.05) and day 4 (*P* < 0.01). At day 1, a higher IL‐10 level was induced in response to CeONP‐3 than to CeONP‐2 (*P* < 0.05), and at day 4, a lower IL‐10 level was obtained with CeONP‐1 than with CeONP‐0 (*P* < 0.05). Therefore, an increase in surface Ce^4+^ content in the CeONPs suppresses the production of a proinflammatory cytokine but induces higher levels of an antiinflammatory cytokine; these are both characteristic responses of M2 macrophages.

## 3 Discussion

**Scheme 1:**
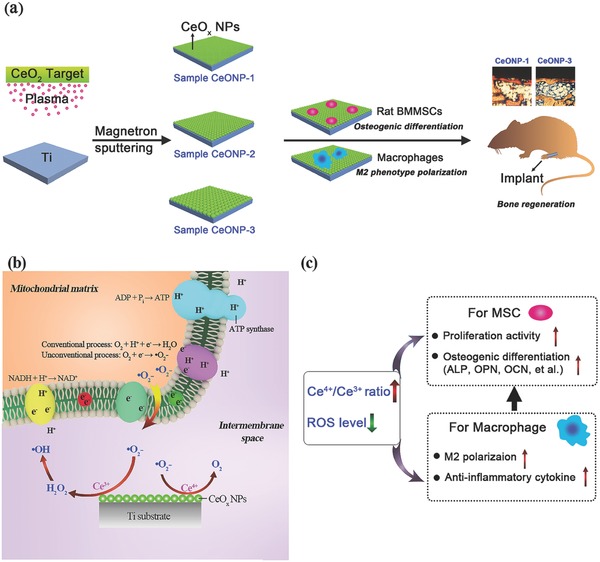
Schematic illustration of CeONPs immobilization on titanium implant biomaterials for bone tissue engineering and regenerative medicine: a) a layer of CeONPs surface fabricated on titanium surface by using magnetron sputtering is able to induce osteogenic differentiation of rat BMSCs and M2 phenotype polarization of macrophages, thereby resulting to the boost of new bone regeneration in vivo; b) the interactions of CeONPs with the superoxide anion radicals (^•^O_2_^−^) produced by mitochondria, including SOD mimetic and catalase mimetic activities; c) the correlations of valence state of CeONPs (Ce^4+^/Ce^3+^ molar ratio) layer with the cell fates of BMSCs and macrophages on osteogenesis.

During magnetron sputtering using highly pure CeO_2_ target (**Scheme** **1**a), subtle fluctuations in deposition may cause a dynamic departure from stoichiometry, which then induces a localized cerium or oxygen excess and creates oxygen or cerium vacancy point defects.^34^ In the case of localized oxygen excess, the charge neutrality demands the creation of cerium vacancies (defect reaction in Kröger–Vink notation: O ↔ V_Ce_^4^′ + 4h^•^ + O_O_^×^). The holes (h^•^) are trapped at the nearest‐neighbor Ce^4+^ sites to create Ce^5+^. However, this happens only with difficulty since the highest oxidation state is 4^+^ for cerium. In the case of localized cerium excess, charge neutrality demands the creation of oxygen vacancies (Kröger–Vink notation: O_O_^×^ ↔ V_O_^••^ + 2e′ + 1/2O_2_). The electrostatic attractive forces can trap the electrons (e′) at the Ce^4+^ sites to create Ce^3+^. As a result, the heterogeneous deposition of cerium and oxygen atoms can create an oxygen‐deficient nonstoichiometric phase (CeO_2−_ *_x_*) with oxygen vacancies.

The relative content of Ce^4+^ or Ce^3+^ is a function of the particle size of CeONPs. In general, the fraction of Ce^3+^ in particles increases as the particle size decreases; i.e., the Ce^4+^/Ce^3+^ ratio increases with increases in the particle size.^29^ Here, by tuning the deposition time of the magnetron sputtering, the particle size was controlled and a high Ce^4+^/Ce^3+^ ratio was obtained for the CeONPs (Scheme 1a). XPS analysis revealed the changes in the surface element compositions of the various samples, as shown in Table 1. The development of surface oxygen content serves as an indicator of the change in the surface oxygen vacancy concentration in the various samples. The apparent drop in the surface oxygen content from 74.47 at% for CeONP‐0 to 70.93 at% for CeONP‐1 indicated the presence of oxygen vacancies on the surface. Subsequent increases in the surface oxygen content revealed a decrease in the number of oxygen vacancies on the surface, which corresponded to the increase in surface Ce^4+^ content and the decrease in surface Ce^3+^ content in the CeONPs. The sampling depth of XPS analysis is ≈10 nm,^35^ so the measured Ce^4+^ and Ce^3+^ contents were derived from the outermost surface of the CeONPs. With regard to the bonding between CeNPs and titanium substrate, only the diffraction peaks of CeO_2_, TiO_2_, and Ti phases are detected in XRD (Figure 1e). Also, all the XPS spectra of Ce3d core levels in Figure 1g–j belong to the cerium oxide, i.e., CeO_2_ or Ce_2_O_3_ and all the XPS spectra of Ti2p core levels in Figure 1n–q are labeled to TiO_2_. Hence, no interfacial reaction is found between cerium oxide and titanium oxide on the titanium substrate.^36^ Therefore, we believe that the cerium oxides are physically bonded to the titanium surface during magnetron sputtering.

The Ce^4+^ state is the preferentially formed (CeO_2_) oxide of cerium. Nevertheless, intrinsic defects are usually present, so that cerium will be present, in part, in the Ce^3+^ state (Ce_2_O_3_) containing oxygen vacancies.^37^ The relative contents of Ce^3+^ and Ce^4+^ are a function of the particle size of the CeONPs,^15^, ^29^ such that the proportion of Ce^3+^ in the particles increases with decreasing particle size. The particle size of CeONPs can be regulated by magnetron sputtering and vacuum annealing, and the vacuum annealing can cause the deposited cerium oxide to grow into nanoparticles via Ostwald ripening. An increase in deposition time can further contribute to an increase in the particle size of the CeONPs.^38^ The relative contents of surface Ce^3+^ and Ce^4+^ in the CeONPs were manipulated in this way in the present study, as shown in Figure 1k. The calculated surface Ce^4+^/Ce^3+^ ratios for the CeONPs were 0.46, 1.23, and 3.23 for CeONP‐1, CeONP‐2, and CeONP‐3, respectively. Therefore, the prepared CeONPs were in a mixed valence state with an increasing surface Ce^4+^/Ce^3+^ ratio. As shown in Figure 2, an increase in the surface Ce^4+^/Ce^3+^ ratio can promote the proliferation of rat BMSCs on CeONPs. This agrees with previous work showing that the nano‐CeO_2_ region on a polymer scaffold promoted cell proliferation, while the nano‐Ce_2_O_3_ region inhibited cell proliferation.^20^

Figure 2 shows that the prepared CeONPs samples had favorable cytocompatibility. The in vivo animal test performed to investigate the influence of surface Ce^4+^/Ce^3+^ ratios on bone formation and mineralization around the CeONP‐immobilized implants confirmed that the extent of new bone tissue formation and mineralization depended on the surface Ce^4+^/Ce^3+^ ratio of the CeONPs (Scheme 1a).

The micro‐CT, sequential fluorescent labeling, and histological analysis results presented in Figure 3 further confirm that an increase in the surface Ce^4+^/Ce^3+^ ratio can promote new bone formation and mineralization around a CeONP‐immobilized implant. The better outcome for the biomechanical push‐out test, as shown in Figure 4, also demonstrates the increasing osseointegration quality of the implant with the increasing surface Ce^4+^/Ce^3+^ ratio. These results were further supported by the SEM morphology observations and EDS element mapping of the newly formed bone tissues around the CeONPs‐immobilized implants, as shown in Figure 5 and Figure S1 (Supporting Information). The mechanism by which the Ce^4+^/Ce^3+^ ratio on the CeONP surface modulates new bone formation and mineralization was investigated by analyzing the expression levels of the osteogenesis‐related genes, ALP, Col‐I, OCN, OPN, and Runx‐2, by quantitative real‐time PCR in in vitro cultured rat BMSCs treated with various CeONP samples. The expression of these markers, as shown in Figure 6a–j, indicated an apparent relationship between the osteogenic differentiation of rat BMSCs and the surface Ce^4+^/Ce^3+^ ratio of the CeONPs.

An increase in the surface Ce^4+^/Ce^3+^ ratio promoted the in vitro osteogenic differentiation of rat BMSCs cultured on CeONPs. This response was also evident at the protein level, as shown in Figure 6k–m. SOD is an enzyme that catalyzes the disproportionation of superoxide, the most common free radical in body, into H_2_O_2_ and O_2_.[[qv: 23b]] The Ce^4+^/Ce^3+^ valence switch capability can endow CeONPs with SOD mimetic activity. The CeONPs with lower Ce^4+^/Ce^3+^ ratio have a higher SOD mimetic activity, and the superoxide dismutation by CeONPs is expressed as^24^

(1) O•2− + Ce3+ + 2H+ → H2O2 + Ce4+; O•2− + Ce4+ → O2 + Ce3+

Catalase is a protective enzyme found within almost all living organisms exposed to oxygen. This enzyme catalyzes the degradation of H_2_O_2_, a powerful and potentially harmful oxidizing agent, into H_2_O and O_2_.^39^ CeONPs can possess catalase mimetic activity that appears in a redox‐state‐dependent manner, and a higher Ce^4+^/Ce^3+^ ratio gives a higher catalase mimetic activity.^25^

Interestingly, the SOD mimetic activity and catalase mimetic activity of CeONPs are the opposite of that expected based on the Ce^4+^/Ce^3+^ ratio (Scheme 1b). CeONPs showing SOD mimetic activity will generate H_2_O_2_. Both in vitro and in vivo, excess H_2_O_2_ is believed to be more toxic than superoxide because it is the substrate for the Fenton reaction that creates the hydroxyl radical (•OH), the most destructive of the ROS.^40^ Fortunately, CeONPs have both SOD and catalase mimetic activities, so the H_2_O_2_ created during the SOD mimetic process can enter into the catalase mimetic dismutation cycle and be decomposed into innocuous H_2_O and O_2_; these reactions make CeONPs an excellent antioxidant.^21^, ^26^ Nevertheless, the antioxidative function of the CeONPs is only effective when the two enzyme mimetic activities are coordinated; in other words, the H_2_O_2_ decomposition rate should be equal to or higher than the production rate.

The SOD mimetic and catalase mimetic activities of the CeONPs should have a close relationship with the osteogenic differentiation of rat BMSCs, since these enzyme mimetic activities can regulate the production of ROS (Scheme 1c). The overproduction of ROS will decrease the osteogenesis of stem cells, but exogenous antioxidant treatment will promote this osteogenesis.^41^ This dichotomy may account for the promoting effect observed for an increase in the surface Ce^4+^/Ce^3+^ ratio with respect to the in vitro osteogenic differentiation of rat BMSCs cultured on CeONPs. A recent study using cerium oxide nanoparticles for functional recovery of spinal cord injury (SCI) indicated that the nanoparticles had a higher +4 oxidation status than +3 (Ce^4+^/Ce^3+^ = 2.9) and these CeONPs possibly carried out an effective consumption of ROS to give rise to SCI recovery at the cost of changing their status primarily from Ce^4+^ to Ce^3+^.^19^

The host immune response is an essential component of the biomaterial‐mediated osteogenic effect.[[qv: 1b]] Therefore, murine RAW264.7 macrophages were cocultured with CeONP samples to obtain a further understanding of the relationship between the surface Ce^4+^/Ce^3+^ ratio of the CeONPs and new bone formation and mineralization. As shown in Figure 7, an increase in the surface Ce^4+^/Ce^3+^ ratio of the CeONPs promoted macrophage polarization to the M2 phenotype and an increase in the production of the anti‐inflammatory cytokine IL‐10. Similarly, a decrease in the surface Ce^4+^/Ce^3+^ ratio promoted macrophage polarization to the M1 phenotype and increased the production of the proinflammatory cytokine TNF‐α. Taken together, these findings indicate that manipulation of the surface Ce^4+^/Ce^3+^ ratio of the CeONPs can modulate the balance of anti‐inflammatory and proinflammatory cytokines in macrophages and create an anti‐inflammatory microenvironment (Scheme 1c).

Interestingly, previous work that investigated the capability of CeONPs to scavenge ROS and inhibit inflammatory mediator production in murine J774A.1 macrophages indicated that oxidative stress and proinflammatory iNOS protein expression were abated by CeONP stimulation.^26^ More importantly, recent work has shown that the prohealing M2‐polarized macrophage phenotype can elicit positive outcomes, both in vitro and in vivo, on osteogenesis, angiogenesis, and osseointegration.[[qv: 8a,10]] The CeONPs appear to show a valence‐dependent modulatory effect on macrophage polarization and cytokine production. In this context, in the present study, CeONP‐1 had a negative effect on the balance between antiinflammatory and proinflammatory macrophage polarization and cytokine secretion, and its outcome was poorer even than that of CeONP‐0.

The long‐term survival and integration of biomaterials are largely determined by activation of the host immune system.[[qv: 1b,42]] Once a biomaterial is implanted into the host body, a sequence of immune responses and healing processes will occur in the surrounding tissues.[[qv: 1b,42a]] Macrophages play a vanguard role in the recognition of and adhesion to the foreign biomaterial.[[qv: 42b,43]] The close relationship between the immune and skeletal systems means that activated macrophages contribute to both the success and the failure of transplantation of a foreign biomaterial. The macrophages exert this dichotomous effect by secreting cytokines that modulate either osteogenesis or inflammation, thereby promoting or inhibiting new bone formation.[[qv: 42b,44]]

A favorable macrophage polarization can create an osteogenic microenvironment that improves osteogenesis, whereas an unfavorable macrophage polarization may exacerbate inflammation and destroy the tissue–biomaterial integration.^12^, ^45^ In general, the M2 macrophage phenotype accounts for antiinflammation and tissue regeneration, whereas the M1 macrophage phenotype is proinflammatory and causes tissue destruction. The results presented here indicate that an increase in the surface Ce^4+^/Ce^3+^ ratio of the CeONPs can promote the polarization of the healing‐associated M2 macrophage phenotype and increase the secretion of antiinflammatory cytokine IL‐10. The antiinflammatory cytokines secreted by M2 macrophages can resolve inflammation and promote wound healing. IL‐10 can inhibit pro‐inflammatory cytokine secretion and activity and the secretion of granulocyte‐macrophage colony stimulating factor and nitric oxide (NO) in macrophages.^46^ By contrast, the secretion of pro‐inflammatory cytokines by M1 lead to delayed bone healing and pathogenic bone loss.[[qv: 42b,43]] As a result, the inhibition of TNF‐α secretion by an increased surface Ce^4+^/Ce^3+^ ratio would benefit new bone formation and wound healing around a CeONP implant.

In summary, the mechanism of surface Ce^4+^/Ce^3+^ ratio of CeONPs on titanium substrate to induce bone‐material integration is illustrated by a schematic diagram (Scheme 1). Manipulating the surface Ce^4+^/Ce^3+^ ratio of CeONPs can modulate macrophage polarization and cytokine secretion, and facilitate appropriate immune reactions that balance antiinflammation and pro‐inflammation, which lead to the satisfactory outcomes of new bone formation and bone–biomaterial integration. The introduction of a biomaterial into the body initiates an inflammatory cascade due to cell and tissue damage, and this cascade persists for roughly 4 d.^13^ This early inflammatory response is highly beneficial to the host, it must subsequently be terminated to avoid tissue damage and to promote tissue regeneration.^14^ Any unrestrained pro‐inflammatory M1 macrophage polarization induced by biomaterials will therefore impair new bone formation and osseointegration[[qv: 8b]] and prevent wound healing.^47^ Therefore, CeONPs biomaterials must have appropriate modulatory effects on the balance between antiinflammation and pro‐inflammation immune reactions in order to elicit the desired outcomes of bone regeneration and osseointegration.

## 4 Conclusion

A customized magnetron sputtering and vacuum annealing protocol is applied to establish a layer of cerium oxide nanoparticles (CeONPs) with different surface Ce^4+^/Ce^3+^ ratios (0.46, 1.23, 3.23) on titanium surface serving as an experimental platform to examine the regulatory effect of CeONPs on cell fate and bone formation. The CeONPs with a mixed valence state and a high surface Ce^4+^/Ce^3+^ ratio exhibit better cytocompatibility with rat BMSCs. Moreover, when implanted into the rat femurs, it is found that new bone formation and bone–implant integration is highly correlated with the surface Ce^4+^/Ce^3+^ molar ratio. The in vivo outcomes are supported by the in vitro studies of rat BMSCs cultured with CeONP samples. The results confirmed that the osteogenic related gene and protein expressions were significantly upregulated, when the cells cultured with the titanium surface with higher Ce^4+^/Ce^3+^ ratio. Furthermore, while culturing with RAW264.7 murine macrophages, the polarization of macrophages to the M2 phenotype is highly expressed on the surface with increased Ce^4+^/Ce^3+^ ratio in which the gain of prohealing M2 percentage can increase antiinflammatory cytokine production. Thus, the mixed valence state of CeONPs has the potential to induce bone regeneration without the need for any exogenous osteogenic inducer. These results suggest that manipulation of the valence state of CeONPs can exert a desirable modulatory effect on stem cell and macrophage fates to elicit the beneficial outcomes of CeONPs on new bone formation and osseointegration.

## 5 Experimental Section

*Sample Fabrication*: The cerium oxide nanoparticles were deposited with a magnetron sputtering apparatus (ULVAC Corp., Model ACS‐4000‐C4) using a high purity CeO_2_ target at a radio frequency power of 80 W. Acid cleaned metal titanium plates or rods (99.95 wt%) were chosen as the deposition substrate. In brief, titanium plates (10 mm × 10 mm × 1 mm, 20 mm × 20 mm × 1 mm, and 20 mm × 10 mm × 1 mm) and rods (Ø 2 mm × 7 mm) were first pickled in oxalic acid solution (5 wt%) at 100 °C for 2 h to clean the surfaces, followed by thorough washing with ethanol and fresh water. Before CeO_2_ deposition, the deposition chamber was first pumped down to ≈10^−4^ Pa and then pure Ar gas (99.999%) was introduced at 50 sccm. During deposition, the substrate was rotated along the vertical axis at a speed of 10 rpm to improve homogeneity. The samples were fabricated by depositing CeO_2_ on the substrate target by sputtering for 2, 3, and 5 min. The samples were then vacuum annealed at 450 °C. For simplicity, the obtained cerium oxide samples were designated as “CeONP‐1,” “CeONP‐2,” and “CeONP‐3,” respectively. The acid‐cleaned and vacuum‐annealed metal titanium acted as the control material and was designated as “CeONP‐0.”

*Sample Characterization*: The surface morphologies were studied using field‐emission SEM (LEO 1530, Germany); the instrument was capable of EDS. The crystallinity was studied by XRD (Rigaku Ultima IV, Japan) using a Cu Kα (λ = 1.541 Å) source in the range of 2θ = 20°–90° with a glancing angle of 1°. Phase identification was done with the help of the JCPDS database. The surface chemical composition and chemical state were determined by XPS (Physical Electronics PHI 5802) using an Al Kα source (1486.6 eV). The optical diffuse reflectance spectra were recorded on UV–vis–NIR spectrophotometer (Model UV‐4100, Hitachi Corp.).

*Cytocompatibility Evaluation: Cell Culture*: The rat BMSCs were purchased from the Cell Bank of Chinese Academy of Sciences and cultured in Dulbecco's modified Eagle's medium (DMEM; HyClone) supplemented with 10% fetal bovine serum (FBS; Gibco) and a 1% antibiotic/antimyotic solution. The BMSCs were cultured at 37 °C in a humidified 5% CO_2_ incubator and passaged every 3 d at ≈80% confluence. Only the confluent BMSCs at passages 3–5 were harvested for further study.

*Cell Proliferation Assay*: The proliferation activities of rat BMSCs on various samples were determined using the CCK8 assay. Initially, 5 × 10^4^ cells were seeded into the wells of a 24 well plate. After 1, 4, and 7 d of culture, CCK8 solution, at a volume of ≈10% of the culture medium, and incubated for 1 h at 37 °C to react with cells. The medium was refreshed and the absorbance was measured at 450 nm on an ELX ultra microplate reader (BioTek, Winooski, VT). Four samples were used for each group and all tests were repeated three times.

*In Vivo Animal Experiment: Surgical Procedures*: All experimental protocols concerning animals were approved by the Animal Committee of the Ninth People's Hospital Affiliated to Shanghai Jiao Tong University School of Medicine. A rat femoral model was used in this study. Thirty‐two six‐month‐old female Sprague–Dawley (SD) rats were randomly divided into the following four groups (8 rats/group): (i) CeONP‐0 group (*n* = 8), (ii) CeONP‐1 group (*n* = 8), (iii) CeONP‐2 group (*n* = 8), and (iv) CeONP‐3 group (*n* = 8). Surgical procedures were performed under sterile conditions, as described previously.^48^ Briefly, the rats were first anesthetized by intraperitoneal injection of ketamine. After the hind limb was shaved, an ≈0 mm longitudinal incision was made across the knee joint along the lateral side of the extensor mechanism. A pilot hole was drilled along the long axis of the femur through the intercondylar notch and the distal femoral metaphysis, and the implants were inserted. After surgery, all rats received antibiotic and analgesic injections intramuscularly for three postoperative days.

*Sequential Fluorescent Labeling*: A polychrome sequential fluorescent labeling method was used to assess the process of new bone formation and mineralization.^48^ At 2, 4, and 6 weeks after surgery, different fluorochromes were intraperitoneally administered at a sequence of 30 mg kg^−1^ Alizarin Red S (Sigma), 25 mg kg^−1^ tetracycline hydrochloride (Sigma), and 20 mg kg^−1^ calcein (Sigma), respectively.

*Sample Preparation*: All rats were sacrificed at weeks 8 after surgery. Left femurs, with four groups of implants, were harvested and trimmed into smaller samples for subsequent use (i.e., 8 left femurs/group, *n* = 8).

*Micro‐CT Evaluation*: The presence of newly formed bone around the inserted implants was determined using micro‐CT (GE explore Locus SP Micro‐CT, USA). The scanning parameters were set at 80 kV and 80 µA, with 3000 ms of exposure time and 15 µm of resolution. After scanning, 3D images were reconstructed using NRecon software (SkyScan, USA) and the CTvol program (SkyScan). The BMD, bone volume fraction (bone volume/total volume, BV/TV), trabecular number (Tb.N), and trabecular thickness (Tb.Th) were analyzed for newly grown bone tissues using DataViewer software (SkyScan) and the CTAn program (SkyScan).

*Histomorphometric and Histological Observation*: After micro‐CT scanning, the femur samples of each group were dehydrated in a graded series of alcohol solutions from 75% (v/v) to 100% (v/v), and embedded in polymethylmethacrylate. The samples were cut into 150 µm thick sections using a Leica SP1600 saw microtome (Leica, Nusseloch, Germany). These sections were then ground and polished to a final thickness of ≈40 µm for fluorescence labeling observation with a confocal laser scanning microscope (CLSM; Leica TCS Sp2 AOBS, Germany). Excitation/emission wavelengths for the chelating fluorochromes were 405/580 nm for tetracycline hydrochloride (yellow), 543/617 nm for Alizarin Red S (red), and 488/517 nm for calcein (green). The percentage of each single fluorochrome staining area, indicating the new bone formation and mineralization at 2, 4, and 6 weeks after surgery, was calculated with Image‐Pro Plus software (Media Cybernetics, Silver Spring, MD, USA). After fluorescence analysis, these sections were stained with Van Gieson's picrofuchsin for histological observation and histomorphometric analysis.

*Push‐Out Test*: This biomechanical test was conducted on a universal material testing system (Instron, High Wycombe, UK). A special custom‐designed holder was used for the test samples to ensure the test force was along the long axis of the implants, which were trimmed to fit into the holder. All tests were conducted at a loading rate of 5 mm min^−1^. The load–displacement curves were recorded during the pushing period. The failure load was defined as the peak load value of the load‐displacement curve.

*SEM Observation*: Rat femurs with four groups of implants were randomly selected for SEM observation. Briefly, after the push out tests, the implants were fixed with 2.5% glutaraldehyde solution at 4 °C overnight, and then sequentially dehydrated in a graded series of ethanol solutions (30, 50, 75, 90, 95, and 100%, v/v). Prior to SEM observation, the samples were sputter coated with platinum. The corresponding elemental distribution on the implant surfaces was detected by EDS mapping.

*In Vitro Osteogenic Evaluation: ALP Activity Assay*: Rat BMSCs were seeded in 24 well plates at density of 5 × 10^4^ cells per well. After 7 d of culture, the cells were stained using an ALP kit (Beyotime, China), according to the manufacturer's instructions. For ALP quantitative assay, the cells were incubated with p‐nitrophenyl phosphate (Sigma) at 37 °C for 30 min. ALP activities were determined by recording optical density (OD) values at 405 nm. Total protein contents were calculated using a Bio‐Rad protein assay kit (Bio‐Rad, USA) and normalized with a range of bovine serum albumin (BSA) (Sigma) standards at 630 nm. ALP activities were expressed as OD values at 405 nm per mg of total protein.

*Calcium Deposition Assay*: Calcium deposition was evaluated at 14 d by ARS staining. Cells were washed twice with phosphate buffered saline (PBS) and fixed in 95% alcohol for 15 min. The cells were stained with 0.1% ARS solution and then desorbed with 10% cetylpyridinium chloride (Sigma) for quantification. The OD values for absorbances of the eluent were recorded at 590 nm. Total protein contents were measured using the Bio‐Rad protein assay kit at 630 nm. Results were normalized and expressed as OD values per mg of total protein.

*Real‐Time PCR Analysis*: Total cellular RNA was extracted at 1 and 7 d with TRIzol reagent (Invitrogen) according to the manufacturer's instruction. Two micrograms of total RNA was used as the template for reverse transcription with Prime‐Script RT reagent kit (Takara Bio, Shiga, Japan). The expression of osteogenic genes, including Runx‐2, OCN, OPN, Col‐I, and ALP, was determined using a real‐time PCR system (Bio‐Rad) with SYBR GREEN PCR Master Mix. The primer sequences for these genes are listed in Table S1 (Supporting Information). Gene‐specific primers were synthesized commercially (Shengong Co., Ltd. Shanghai, China). The housekeeping gene, glyceraldehyde‐3‐phosphate dehydrogenase (GAPDH), was used for normalization. CeONP‐0 acted as the control for relative gene expression. All assays were carried out in triplicate.

*Immunofluorescence Observation*: OPN expression was detected by seeding rat BMSCs on samples at a density of 5 × 10^5^ cells per well and cultured for 7 d. The samples were then washed with PBS three times and fixed in 4% paraformaldehyde at 4 °C for 30 min. Subsequently, the cells were permeabilized with 0.1% Triton X‐100 for 30 min and blocked in 10% goat serum for 1 h at room temperature. A specific primary antibody targeting rat OPN (Abcam) was added at 1:100 dilution for overnight coincubation at 4 °C. DyLight 488‐conjugated anti‐mouse IgG antibody (Boster, China) at a 1:100 dilution was added and incubated for 30 min at room temperature in the dark. These samples were observed by CLSM after the cell nuclei were contrast‐labeled with DAPI (Sigma, USA).

*Macrophage Study: Cell Culture*: The RAW264.7 murine‐derived macrophage cell line was purchased from the Cell Bank of Chinese Academy of Sciences and maintained in DMEM supplemented with 10% FBS and 1% penicillin/streptomycin at 37 °C in humidified 5% CO_2_ incubator. The culture medium was exchanged every 48 h. The growing cells were passaged at ≈80% confluence by scraping, and only passages 3–5 were used in the study.

*Flow Cytometry Analysis*: Flow cytometry was used for quantitative analysis of the expression of CCR7 (M1 marker) and CD206 (M2 marker). In total, 5 × 10^5^ cells were seeded onto various samples (20 mm × 20 mm × 1 mm). After culturing for 4 d, the cells were trypsinized and scraped from the sample surfaces, centrifuged, and resuspended in 1% BSA for 30 min at ambient temperature to block nonspecific antigens. The cells were then incubated with fluorescein isothiocyanate (FITC)‐conjugated anti‐mouse F4/80, allophycocyanin (APC)‐conjugated CCR7, and phycoerythrin (PE)‐conjugated CD206 for 1 h in the dark at ambient temperature in a final volume of 100 µL. FITC‐conjugated rat IgG2a,κ, APC‐conjugated rat IgG2a,κ, and PE‐conjugated rat IgG2a,κ acted as isotype controls. All flow cytometry antibodies were purchased from eBioscience. After washing twice in 1% BSA, the cells were resuspended in 1% BSA and analyzed with a Guava flow cytometer (Millipore, USA); 5 × 10^3^ cells were analyzed in each test. Data were analyzed using guavaSoft 3.1.1 software.

*Cytokine Measurement*: The production of relevant cytokines was measured using enzyme‐linked immunosorbent assays (ELISAs). After 1 and 4 d of culture, the culture medium was aspirated and centrifuged, and the supernatant was utilized for analyses. The concentrations of TNF‐α and IL‐10 were measured with ELISA kits (Anogen, Canada), following the manufacturer's instructions.

*Statistical Analysis*: All data were expressed as mean ± standard deviation. Statistically significant differences (*P*) among groups were measured by one‐way analysis of variance (ANOVA) and SNK post hoc analysis, based on normal distribution and equal variance assumption test. All statistical analyses were performed using SPSS v.10.1 software (IBM SPSS, Armonk, New York, USA). A value of *P* < 0.05 was considered statistically significant.

## Conflict of Interest

The authors declare no conflict of interest.

## Supporting information

Supplementary

## References

[1] a) R. A. Marklein , J. A. Burdick , Adv. Mater. 2010, 22, 175; 20217683 10.1002/adma.200901055

[2] a) X. Wang , X. Hu , N. Kawazoe , Y. Yang , G. Chen , Adv. Funct. Mater. 2016, 26, 7634;

[3] a) Y. Wu , S. Zhu , C. Wu , P. Lu , C. Hu , S. Xiong , J. Chang , B. C. Heng , Y. Xiao , H. W. Ouyang , Adv. Funct. Mater. 2014, 24, 4473;

[4] P. Yu , C. Ning , Y. Zhang , G. Tan , Z. Lin , S. Liu , X. Wang , H. Yang , K. Li , X. Yi , Y. Zhu , C. Mao , Theranostics 2017, 7, 3387. 28900517 10.7150/thno.19748 PMC5595139

[5] D. C. F. Wieland , C. Krywka , E. Mick , R. Willumeit‐Römer , R. Bader , D. Kluess , Acta Biomater. 2015, 25, 339. 26192999 10.1016/j.actbio.2015.07.021

[6] H. Cao , L. Feng , Z. Wu , W. Hou , S. Li , Y. Hao , L. Wu , Mater. Sci. Eng., C 2017, 80, 7. 10.1016/j.msec.2017.05.078 28866219

[7] C. Ning , P. Yu , Y. Zhu , M. Yao , X. Zhu , X. Wang , Z. Lin , W. Li , S. Wang , G. Tan , Y. Zhang , Y. Wang , C. Mao , NPG Asia Mater. 2016, 8, e243. 27818718 10.1038/am.2016.9 PMC5091659

[8] a) C. Wu , Z. Chen , Q. Wu , D. Yi , T. Friis , X. Zheng , J. Chang , X. Jiang , Y. Xiao , Biomaterials 2015, 71, 35; 26318815 10.1016/j.biomaterials.2015.08.027

[9] a) A. Mantovani , A. Sica , S. Sozzani , P. Allavena , A. Vecchi , M. Locati , Trends Immunol. 2004, 25, 677; 15530839 10.1016/j.it.2004.09.015

[10] a) B. Li , H. Cao , Y. Zhao , M. Cheng , H. Qin , T. Cheng , Y. Hu , X. Zhang , X. Liu , Sci. Rep. 2017, 7, 42707; 28198427 10.1038/srep42707 PMC5309879

[11] a) A. Boddupalli , L. Zhu , K. M. Bratlie , Adv. Healthcare Mater. 2016, 5, 2575; 10.1002/adhm.201600532 27593734

[12] S. Franz , S. Rammelt , D. Scharnweber , J. C. Simon , Biomaterials 2011, 32, 6692. 21715002 10.1016/j.biomaterials.2011.05.078

[13] a) L. Claes , S. Recknagel , A. Ignatius , Nat. Rev. Rheumatol. 2012, 8, 133; 22293759 10.1038/nrrheum.2012.1

[14] C. Nathan , Nature 2002, 420, 846. 12490957 10.1038/nature01320

[15] C. Xu , X. Qu , NPG Asia Mater. 2014, 6, e90.

[16] J. Chen , S. Patil , S. Seal , J. F. McGinnis , Nat. Nanotechnol. 2006, 1, 142. 18654167 10.1038/nnano.2006.91

[17] F. Pagliari , C. Mandoli , G. Forte , E. Magnani , S. Pagliari , G. Nardone , S. Licoccia , M. Minieri , P. Di Nardo , E. Traversa , ACS Nano 2012, 6, 3767. 22524692 10.1021/nn2048069

[18] M. Das , S. Patil , N. Bhargava , J.‐F. Kang , L. M. Riedel , S. Seal , J. J. Hickman , Biomaterials 2007, 28, 1918. 17222903 10.1016/j.biomaterials.2006.11.036 PMC1913191

[19] J.‐W. Kim , C. Mahapatra , J.‐Y. Hong , M. S. Kim , K. W. Leong , H.‐W. Kim , J. K. Hyun , Adv. Sci. 2017, 4, 1700034. 10.1002/advs.201700034 PMC5644223 29051850

[20] T. Naganuma , E. Traversa , Biomaterials 2014, 35, 4441. 24612920 10.1016/j.biomaterials.2014.01.074

[21] C. Mandoli , F. Pagliari , S. Pagliari , G. Forte , P. Di Nardo , S. Licoccia , E. Traversa , Adv. Funct. Mater. 2010, 20, 1617.

[22] C. Xu , Y. Lin , J. Wang , L. Wu , W. Wei , J. Ren , X. Qu , Adv. Healthcare Mater. 2013, 2, 1591. 10.1002/adhm.201200464 23630084

[23] a) I. Celardo , M. De Nicola , C. Mandoli , J. Z. Pedersen , E. Traversa , L. Ghibelli , ACS Nano 2011, 5, 4537; 21612305 10.1021/nn200126a

[24] C. Korsvik , S. Patil , S. Seal , W. T. Self , Chem. Commun. 2007, 1056. 10.1039/b615134e 17325804

[25] T. Pirmohamed , J. M. Dowding , S. Singh , B. Wasserman , E. Heckert , A. S. Karakoti , J. E. S. King , S. Seal , W. T. Self , Chem. Commun. 2010, 46, 2736. 10.1039/b922024k PMC3038687 20369166

[26] S. M. Hirst , A. S. Karakoti , R. D. Tyler , N. Sriranganathan , S. Seal , C. M. Reilly , Small 2009, 5, 2848. 19802857 10.1002/smll.200901048

[27] M. Geetha , A. K. Singh , R. Asokamani , A. K. Gogia , Prog. Mater. Sci. 2009, 54, 397.

[28] X. Liu , P. K. Chu , C. Ding , Mater. Sci. Eng., R 2004, 47, 49.

[29] F. Zhang , C.‐H. Chen , J. M. Raitano , J. C. Hanson , W. A. Caliebe , S. Khalid , S.‐W. Chan , J. Appl. Phys. 2006, 99, 084313.

[30] G. Wang , J. Li , K. Lv , W. Zhang , X. Ding , G. Yang , X. Liu , X. Jiang , Sci. Rep. 2016, 6, 31769. 27546196 10.1038/srep31769 PMC4992888

[31] T. Skála , F. Šutara , K. C. Prince , V. Matolín , J. Electron Spectrosc. Relat. Phenom. 2009, 169, 20.

[32] J. Ng , S. Xu , X. Zhang , H. Y. Yang , D. D. Sun , Adv. Funct. Mater. 2010, 20, 4287.

[33] K. I. Vladimir , A. B. Shcherbakov , A. V. Usatenko , Russ. Chem. Rev. 2009, 78, 855.

[34] J. Li , G. Guo , J. Wang , H. Zhou , H. Shen , K. W. K. Yeung , Nanotechnology 2017, 28, 175705. 28367838 10.1088/1361-6528/aa6525

[35] J. Li , H. Zhou , J. Wang , D. Wang , R. Shen , X. Zhang , P. Jin , X. Liu , Nanoscale 2016, 8, 11907. 27240639 10.1039/c6nr02844f

[36] C. Anandan , P. Bera , Appl. Surf. Sci. 2013, 283, 297.

[37] a) C. T. Campbell , C. H. F. Peden , Science 2005, 309, 713;16051777

[38] H. Zhou , X. Cao , M. Jiang , S. Bao , P. Jin , Laser Photonics Rev. 2014, 8, 617.

[39] P. Nicholls , Arch. Biochem. Biophys. 2012, 525, 95. 22326823 10.1016/j.abb.2012.01.015

[40] B. Lipinski , Oxid. Med. Cell. Longevity 2011, 2011, 1. 10.1155/2011/809696 PMC3166784 21904647

[41] a) J. Tan , X. Xu , Z. Tong , J. lin , Q. Yu , Y. Lin , W. Kuang , Bone Res. 2015, 3, 15003; 26273536 10.1038/boneres.2015.3 PMC4413016

[42] a) J. M. Anderson , A. Rodriguez , D. T. Chang , Semin. Immunol. 2008, 20, 86; 18162407 10.1016/j.smim.2007.11.004 PMC2327202

[43] N. Mokarram , R. V. Bellamkonda , Ann. Biomed. Eng. 2014, 42, 338. 24297492 10.1007/s10439-013-0941-0

[44] Z. Chen , C. Wu , Y. Xiao , in The Immune Response to Implanted Materials and Devices: The Impact of the Immune System on the Success of an Implant (Ed: Corradetti B.), Springer International Publishing, Cham 2017, p. 107.

[45] D. F. Williams , Biomaterials 2008, 29, 2941. 18440630 10.1016/j.biomaterials.2008.04.023

[46] D. M. Mosser , J. P. Edwards , Nat. Rev. Immunol. 2008, 8, 958. 19029990 10.1038/nri2448 PMC2724991

[47] A. Sindrilaru , T. Peters , S. Wieschalka , C. Baican , A. Baican , H. Peter , A. Hainzl , S. Schatz , Y. Qi , A. Schlecht , J. M. Weiss , M. Wlaschek , C. Sunderkötter , K. Scharffetter‐Kochanek , J. Clin. Invest. 2011, 121, 985. 21317534 10.1172/JCI44490 PMC3049372

[48] J. Wen , J. Li , H. Pan , W. Zhang , D. Zeng , L. Xu , Q. Wu , X. Zhang , X. Liu , X. Jiang , J. Mater. Chem. B 2015, 3, 4790. 10.1039/c5tb00128e 32262668

