# Supplementary material for: Valence State Manipulation of Cerium Oxide Nanoparticles on a Titanium Surface for Modulating Cell Fate and Bone Formation
Source: Adv Sci (Weinh). 2017 Dec 18;5(2):1700678. doi: 10.1002/advs.201700678 (PMC5827567; doi:10.1002/advs.201700678)
Supplement: Supplementary file 1 — Supplementary [file ADVS-5-1700678-s001.pdf]

Copyright WILEY-VCH Verlag GmbH & Co. KGaA, 69469 Weinheim, Germany, 2017.

ADVANCED SCIENCE
Open Access

Supporting Information

for *Adv. Sci.,* DOI: 10.1002/advs.201700678

Valence State Manipulation of Cerium Oxide Nanoparticles on a Titanium Surface for Modulating Cell Fate and Bone Formation

Jinhua Li, Jin Wen, Bin Li, Wan Li, Wei Qiao, Jie Shen, Weihong Jin, Xinquan Jiang, Kelvin W. K. Yeung,* and Paul K. Chu**

Copyright WILEY-VCH Verlag GmbH & Co. KGaA, 69469 Weinheim, Germany, 2016.

Supporting Information

Valence State Manipulation of Cerium Oxide Nanoparticles on Titanium Surface for Modulating Cell Fate and Bone Formation

*Jinhua Li, Jin Wen, Bin Li, Wan Li, Wei Qiao, Jie Shen, Weihong Jin, Xinquan Jiang *, Kelvin W. K. Yeung *, and Paul K. Chu **

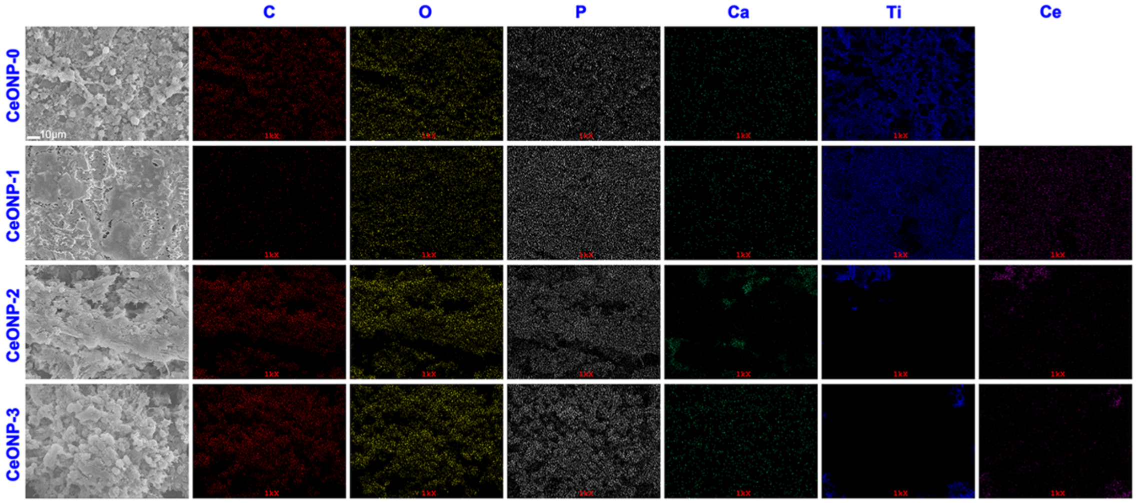

Figure S1: Surface morphologies of the implants of various samples, accompanied by the corresponding EDS elemental mapping and distribution of C, O, P, Ca, Ti and Ce.

Table S1. Primers for real-time polymerase chain reaction (PCR).

Gene	Prime sequence (F, forward; R, reverse)	Product size (bp)	Accession number
GAPDH	F: GGCAAGTTCAACGGCACAGT	76	NM_017008.3
	R: GCCAGTAGACTCCACGACAT		
Runx-2	F: CCGAGACCAACCGAGTCATT	114	NM_001278483.1
	R: CACTGCACTGAAGAGGCTGT		
OCN	F: CAGTAAGGTGGTGAATAGACTCCG	172	NM_013414.1
	R: GGTGCCATAGATGCGCTTG		
OPN	F: CCAAGCGTGGAAACACACAGCC	165	NM_012881.2
	R: GGCTTTGGAACTCGCCTGACTG		
ALP	F: GTCCCACAAGAGCCCACAAT	172	NM_013059.1
	R: CAACGGCAGAGCCAGGAAT		
Col-I	F: CTGCCCAGAAGAATATGTATCACC	198	XM_213440
	R: GAAGCAAAGTTTCCTCCAAGACC		
